# Edible Fungi Are a Hidden Source of Carbon Monoxide and Carbon Dioxide

**DOI:** 10.1111/1462-2920.70259

**Published:** 2026-02-16

**Authors:** Shaojun Xiong, Jannik Demuth, Mohsen Parchami, Geoffrey Daniel

**Affiliations:** ^1^ Department of Forest Bioeconomy and Technology, Biomass Technology and Chemistry Swedish University of Agricultural Sciences Umeå Sweden; ^2^ Department of Forest Bioeconomy and Technology, Wood Science Swedish University of Agricultural Sciences Uppsala Sweden

**Keywords:** biogenesis, carbon emission, edible mushroom, greenhouse gas, working environment

## Abstract

This study provides the first clear evidence that edible mushrooms, such as *Lentinula edodes* (shiitake), *Pleurotus ostreatus* and *Pleurotus eryngii*, can generate carbon monoxide (CO) as part of their metabolic activity—independent of bacteria, illumination or oxygen limitation. Systematic measurements of CO and CO_2_ emissions were performed over 60 days using multiple fungal species, substrates and growth conditions. Microscopy observations (light, scanning and transmission electron microscopy) confirmed no extracellular and intracellular bacterial endosymbionts involved, supporting a fungal genesis of CO. CO emission patterns showed a parabola‐shaped curve, correlating with CO_2_ levels regardless measurements by gas‐analyser or GC–MS and peaking during full mycelial colonisation. Shiitake mushrooms grown on birch substrate released the highest CO compared to alder and aspen substrates and *P. ostreatus* and *P. eryngii*. These findings suggest that fungal respiration contributes to CO dynamics more than previously recognised and highlight the need for further research into its mechanisms and environmental and occupational health implications.

## Graphical abstract

This study provides the first and robust evidence that edible mushrooms, such as *Lentinula edodes*, *Pleurotus ostreatus* and *P. eryngii* , can generate carbon monoxide (CO) as part of their metabolic activity—independent of bacteria, illumination or oxygen limitation. This is largely different from what was previously recognised. The findings resulted from systematic measurements of CO and CO_2_ emissions over 60 days using multiple fungal species, substrates and growth conditions in nine experiments.

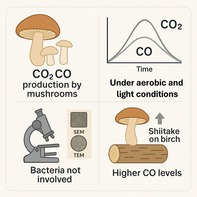

## 1 Introduction

Food production generates more than one‐third of man‐made greenhouse gas emissions (Gilbert 2012; Vermeulen et al. 2012). Approximately 57% of these emissions are attributed to the production of animal‐based food, including meat products, the cultivation of crops for livestock feed and the maintenance of pastures for grazing. Increasing the production of plant‐based protein food may help mitigate the greenhouse gas emissions associated with the food system. In this context, the cultivation of edible mushrooms can be considered a viable alternative strategy. Many edible mushrooms have a high content of protein; for example, up to 21%–27% of protein content (dry mass based) was found in shiitake (*Lentinula edodes*) fruit bodies (United States Department of Agriculture (USDA) 2018; Li et al. 2021). Mushrooms can also offer nutritional and medicinal benefits (Carrasco‐González et al. 2017; Gargano et al. 2017) and be cultivated indoors using local forest and agricultural residues as a sustainable resource.

Recent research and development (Ikeda et al. 2021; Chen, Martín, Finell, et al. 2022; Chen, Martín, Lestander, et al. 2022; Martin et al. 2023) suggest that the cultivation of edible fungi goes beyond the production of protein‐rich and functional food. The cultivation of white rot edible fungi can serve as a starting process for the cascade utilisation of lignocellulose biomass, leading to the production of valuable bio‐based products (Martin et al. 2023). By growing these fungi, waste streams such as forest and crop residues, which serve as the growing substrates, can be converted into high‐quality food that can partially replace meat. Additionally, these fungi secrete enzymes such as lignin‐ and manganese peroxidases and hemicellulases, enabling the selective degradation of lignin and hemicellulose present in the substrates. This process makes the spent substrates less recalcitrant, allowing for the biological conversion of cellulose into sugar platforms, ethanol (Chen, Martín, Finell, et al. 2022; Chen, Martín, Lestander, et al. 2022), biogas (Lin et al. 2015) and biomaterials (Shakir et al. 2023; Zhao et al. 2024). These findings highlight the great potential of integrating edible mushroom cultivation with the production of bio‐based products, contributing to the development of a bioeconomy.

The global production of cultivated edible mushrooms reached 42.8 million tons in 2020, marking a 13.8‐fold increase over the past 30 years (Okuda 2022). The top five mushrooms, namely *L. edodes* (22%), *Pleurotus* spp. (19%), *Auricularia* spp. (18%), *Agaricus bisporus* (15%) and *Flammulina velutipes* (11%), all belong to the group of white rot fungi (Royse et al. 2017). In order to foster continuous development, it is crucial to gain a deeper understanding of the processes involved, not only in meeting global demand but also in ensuring environmental sustainability (Okuda 2022). A significant aspect in this regard is the study of carbon emission fluxes during mushroom cultivation. Edible mushrooms belong to the Basidiomycota fungal group. As they grow, the fungal mycelium degrades the lignocellulose substrate and converts the degraded components into new carbohydrates and proteins in their tissues. Meanwhile, fungal metabolic activities generate significant carbon emissions such as carbon dioxide (CO_2_) (Jung and Son 2021) and methane (CH_4_) (Lenhart et al. 2012; Ernst et al. 2022). These gas emissions, which in particular contribute to greenhouse gas‐induced climate change and CO_2_, may also influence physiological disorders in mushrooms (Donoghue and Denison 1995; Jung and Son 2021).

Compared to the extensive knowledge available on CO_2_ and CH_4_, the emission of CO by fungi remains poorly understood. Existing academic reports on CO biogenesis primarily focus on bacteria as a major origin and suggest that both aerobic and anaerobic bacteria can generate CO during organic decomposition (Sobieraj et al. 2023). Similarly, other organisms, such as seed plants, have also been suggested as sources of CO. The first known report on the biogenesis of CO from edible fungi was by Siegel and Siegel (1987), although Westlake et al. (1961) had earlier reported the mould fungus *Aspergillus flavus* could generate CO when the oxygen supply was limited. In the Siegel and Siegel (1987) study, they observed CO emission from fruit‐body tissues (after ‘washing in distilled water’) of *Agaricus* and *Auricularia* during the initial 3–6 days of incubation in the dark and under hypoxia (5% oxygen) conditions. However, they noted bacterial growth becoming evident after Day 12, leading to a decline in CO levels. This raises questions regarding the involvement of bacteria or other microorganisms in the CO generation process. In a pilot study conducted by the authors of this paper, CO emissions were observed during the cultivation of edible fungi under various conditions, including well‐aerated (~20% oxygen) environments and under light exposure. These findings emphasise the need to understand: (1) the origin of CO, specifically whether it was solely generated by fungi; (2) the biotic and abiotic factors that may influence CO production and (3) the dynamics of CO emissions during fungal cultivation. Addressing question 3 is particularly important from a workplace safety perspective, as CO is considered a toxic gas when its concentration reaches 50 ppm in poorly ventilated areas. Furthermore, CO is considered an indirect greenhouse gas that may impact atmospheric CO_2_, ozone (O_3_) and CH_4_ concentrations in the lower atmosphere (Alakoski et al. 2016). Therefore, it is crucial to evaluate these gas emissions during mushroom cultivation, since the knowledge and data currently available on this topic is limited in the literature.

The development of knowledge on mushroom(s) emission of gases and the corresponding mechanisms involved is dependent on effective measurement methods. In academic research, gas emissions are normally measured through gas chromatography (GC–MS) on a limited set of samples taken. However, for a practical use especially for monitoring the gas emissions for the mushroom production industry, a portable method is needed. Although there are reports on using market‐available probes to measure CO_2_ (Pavlík et al. 2020) as an index of the metabolic activity of white‐rot fungi, its accuracy to analyse CO remains to be confirmed. Implementing simple ways to accurately measure emissions of CO_2_ and other gases (e.g., CO), is important for monitoring and obtaining an insight of fungal respiration during different growth stages and/or lignocellulose degradation.

The primary objective of this study was to investigate, alongside the quantification of CO_2_ emission during fungal cultivation, the potential source of CO emissions by conducting experiments using shiitake mushrooms (*L. edodes*) grown on birch as a substrate model. The experiments were to assess the impact of different factors, including aerobic supply, nitrogen levels, light exposure, substrate variety and fungal species on CO emissions. Two additional fungi, namely *Pleurotus ostreatus* and *Pleurotus eryngii*, and two other wood substrate species, alder and aspen, were also included in the study. Both a portable gas analyser and gas chromatography–mass spectrometry (GC–MS) techniques were employed to compare their sensitivity difference. In all experiments, CO_2_ was analysed together with CO measurements, at regular intervals throughout the entire incubation period that span up to 60 days.

This study is to determine whether edible fungi, using *L. edodes* as a model species, can produce carbon monoxide (CO) as an intrinsic metabolic by‐product, independently of microbial contamination or anaerobic conditions. To test this, we conducted a series of controlled experiments evaluating CO and CO_2_ emissions under varying abiotic (illumination, aeration, nitrogen) and biotic (substrate type, fungal species, inoculum) conditions. By applying both microscopy and gas measurement techniques, we aimed to: (1) Confirm whether CO emission is generated solely by fungal activity; (2) Assess how different environmental and biological factors influence the dynamics of CO release and (3) Evaluate the practical significance of fungal CO emissions for environmental monitoring and workplace safety.

## 2 Materials and Methods

### 2.1 Materials

Sawdust (< 3 mm) of white birch ( *Betula pubescens* Ehrh.), alder ( *Alnus incana* (L.) Moench) and aspen ( *Populus tremula* L.) were used as major ingredients of the mushroom growing substrates. Twenty‐eight percent of either of the three sawdust types was mixed proportionally by weight with 6.7% wheat ( *Triticum aestivum* L.) bran, 0.34% CaCO_3_ and 65% water. The substrate had a pH of approximately 5.95–6.34 after pasteurisation or autoclaving.

### 2.2 Experiments

**FIGURE 1:**
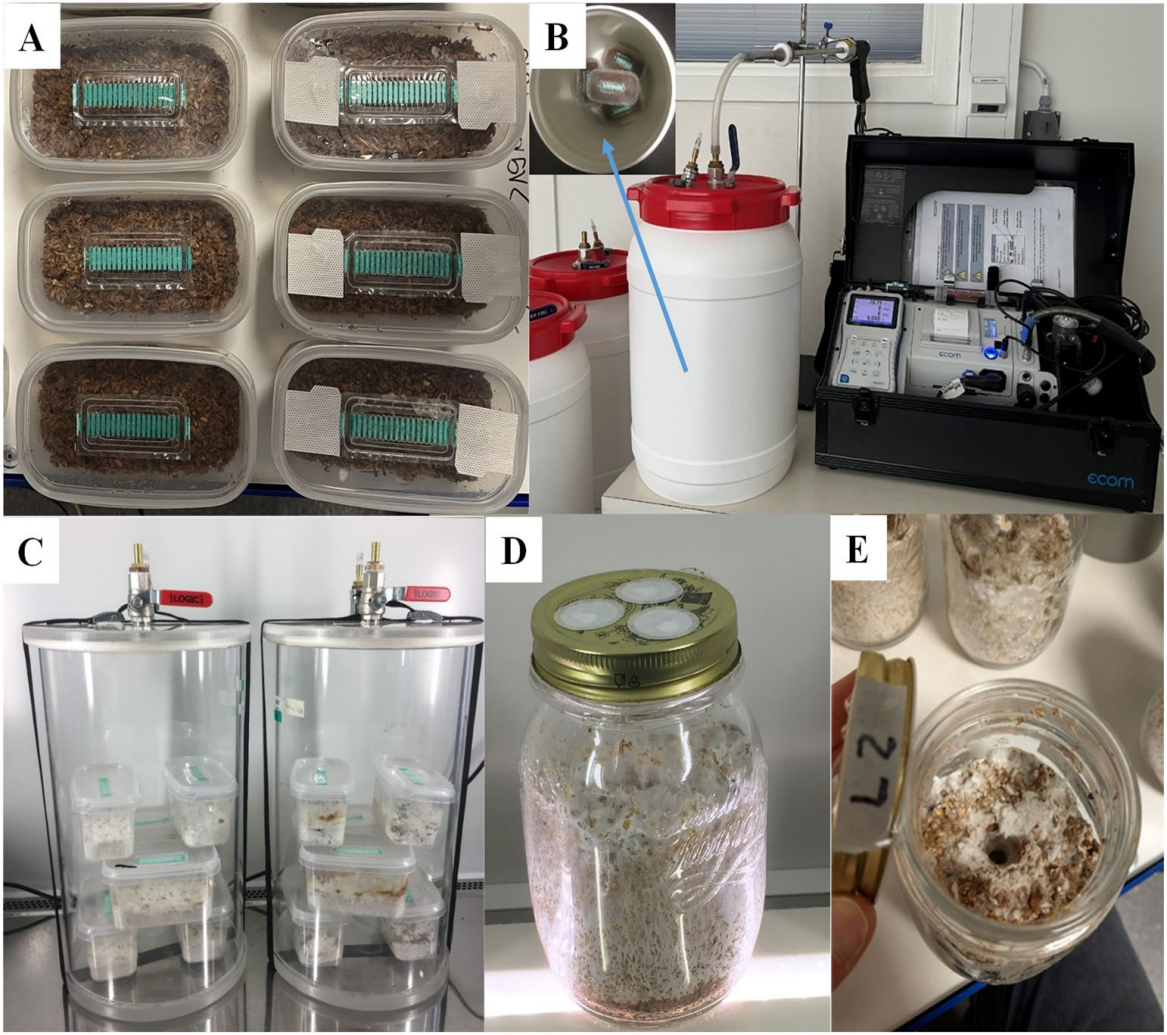
Major components of the experiments. (A) Cultivation container; (B) air‐tight plastic drum and potable gas analyser for gas emission measurements for the treatments without light; (C) enclosed/sealed transparent cylinders containing growing shiitake mycelia under light (Experiment 6a) during gas accumulation before gas emission measurements and (D and E) cultivation jar used for Experiment 6b.

This study includes a series of experiments that are specified in Table 1 and outlined in Figure 1.

**TABLE 1:** Experimental settings and gas parameters.^a^

Experiment/Treatment	Substrate	Pasteurisation	Fungus	Inoculum	Aeration‐filter	Whey	Illumination	Temperature (°C)^b^		Oxygen (%, v/v)^b^	
		/autoclaving		(spawn)		%		Ambient	Gas	Ambient	Gas
1. Non‐inoculated	Birch	85°C hot air	None	Grain	1	0	Dark	20–23	20–25	20.6–21.0	20.4–20.9
		125°C hot air						20–24	21–25	20.6–21.0	20.6–21.0
		121°C steam						22–23	22–23	20.6–21.0	20.6–21.0
2. Pasteurisation	Birch	85°C hot air	*L. edodes*	Grain	1	0	Dark	19–22	20–24	20.9–21.0	19.4–20.6
		125°C hot air						20–24	20–25	20.7–21.0	18.4–20.8
		121°C steam						20–24	21–25	20.6–21.0	20.1–21.0
3. Inoculum type	Birch	121°C steam	*L. edodes*	Grain	1	0	Dark	21–24	22–24	20.6–21.0	19.4–20.9
		(Double)		PDA				22–24	22–24	20.6–21.0	19.7–20.9
4. Aeration	Birch	121°C steam	*L. edodes*	Grain	1	0	Dark	20–23	20–25	20.6–21.0	20.4–20.9
					3			20–23	20–25	20.6–21.0	20.1–21.1
5. Nitrogen addition	Birch	121°C steam	*L. edodes*	Grain	1	0	Dark	18.4–22.4	18.2–22.5	20.6–21.0	18.0–20.8
						2		18.1–22.4	18.2–22.3	20.6–21.0	18.9–20.7
6a. Illumination	Birch	121°C steam	*L. edodes*	Grain	1	0	Light	22–24	22–24	20.6–21.0	19.4–20.9
							Dark	21.5–24	22–24	20.6–21.0	19.8–20.8
6b. Illumination	Birch	121°C steam	*L. edodes*	Grain	1	0	Light	19.9–24.1	18.6–21.3	20.8–21.0	20.3–20.8
(GA vs. GC–MS)^c^		(Double)					Dark	19.9–24.1	19.6–21.1	20.8–21.0	20.3–20.8
7. Substrate variety	Birch	121°C steam	*L. edodes*	Grain	1	0	Dark	19–22	20–24	20.9–21.0	19.4–20.6
	Alder							19–23	19–24	20.6–21.0	19.2–20.7
	Aspen							19–24	20–25	20.6–20.9	19.3–20.7
8. Fungal species	Birch	121°C steam	*L. edodes*	Grain	1	0	Dark	19–22	20–24	20.9–21.0	19.4–20.6
			*P. ostreatus*					21–23	22–24	20.6–21.0	19.6–20.5
			*P. eryngii*					21–23	22–24	20.7–21.0	19.9–20.8

^a^ Gas parameters were ambient before and during gas flux measurements.

^b^ The range (min.–max.) for all measurement occasions during the entire study period.

^c^ GA = gas analyser, GC–MS = gas chromatography mass spectrometry.

For most experiments (except Experiment 6b, see further below), the moisturised substrate was packed into polypropylene container ‘microboxes’ (Figure 1A; 125 × 65 × 80 mm) that were sealed by a lid but equipped with micro‐porous filters for gas exchanges and bio‐filtration on the top of the microbox lid (Microsac, http://saco2.com/). However, to determine the effect of aeration (treatment 4), two extra holes (8 mm in diameter) covered with micro‐porous HEPA filters were made in the lids of the boxes (Figure 1A). Each microbox was filled with 200 g wet substrates (i.e., 70 g DM) and then pasteurised in a hot‐air dryer or sterilised with steam in an autoclave, according to the experiment setting. Commercial grain spawn of *L. edodes* (strain M3790), *P. ostreatus* (M2140) and *P. eryngii* (M2603) were inoculated at a ratio of 2% of wet substrate weight. In Experiment‐3, potato dextrose agar (PDA) inoculum that had been prepared in petri plates were inoculated in four containers, alongside the inoculation with grain spawn for the other four microboxes. The substrates in all eight boxes of Experiment‐3 were double autoclaved, to reduce the possibility of any bacterial contamination. After inoculation, the majority of the microboxes were kept in a dark incubation room even during gas measurements (Figure 1B), except for those for light treatments (Figure 1C) that were placed under light (1032–1321 lx). Incubations were performed at room temperature (21°C–22°C) and relative humidity of 60%–70% for all treatments during the experiment period. Four replicates (microboxes) were used for most experiments except for Experiment 6a in which six replicates were used for light and six for dark, with a thinner layer of the substrate (70 g wet mass) used to increase the total areas receiving light.

### 2.3 Measurement of Gases

Two methods were applied to identify and quantify the gas emissions during the mycelial incubation, namely, GC/MS analysis and gas–metre measurements.

A potable gas analyser (Ecom‐J2KN, rbr Masstechnik GmbH, Germany) was adapted for measuring the gas emissions for all treatments. The same gas analyser had been used by other researchers to measure off‐gas emissions from wood biomass and pellet fuels (Alakoski et al. 2016; Siwale et al. 2024). Gas measurements were conducted at intervals of 3–4 days during the incubation period. Before measurements and for most treatments except for lightness treatment, all four replicated containers of each treatment were collectively placed in an air‐tight plastic 20 L drum, equipped with a lid and a valve on top (Figure 1B). Gas analysis for lightness treatment was performed using a glass cylinder instead (Figure 1C). As soon as the lid and the valve were closed, the gases emitted and accumulated inside the enclosed drum/cylinder. The accumulation duration was between 2 and 4 h depending on the growth stage, which was determined by a pilot study that had showed an optimal duration with no significant air pressure change inside the drum/cylinder. When the accumulation time was completed, the valve was turned on to connect the gas analyser to extract and measure the gas from inside the drum or glass cylinder. Three readings were recorded at 1, 1.5 and 2 min after starting the gas analyser. The ambient gas was also measured before accessing the valve to the gas analyser.

To verify the identification of gases and results from the gas analyser, gas chromatography–mass spectrometry (GC–MS, Clarus 580, PerkinElmer, USA) was applied, which was integrated with an additional Experiment 6b (Table 1 and Figure 1D,E). Glass jars of 600 mL containing 240 g of wet substrate were used for this purpose instead to facilitate the sampling method. The lid of each jar had three 1‐cm holes (in the middle) equipped with filters to allow air exchange during fungal incubation and gas–metre measurements. An approximately 1.5–2.0 cm diameter hole was cut through the middle of the substrate (Figure 1E, from top to bottom) to create more surface area for the light to penetrate and for gas exchange. Three jars (replicates) were placed in the dark and three under light (15,230 lx above and 1032 lx below the jars). When sampling gases for GC–MS analysis, the lids with air‐exchange filters were replaced by an air‐tight lid (with one hole but equipped with a rubber septum) to allow gas accumulation inside the jar. After 2.5‐h accumulation time, an air‐tight syringe (12 mL) was used to extract the gas from the jar by penetrating the needle through the rubber septum on the airtight lid. The gas sample was then injected into a pre‐vacuumed glass vial (12 mL) and stored at room temperature until GC–MS analysis. The samples were then analysed by GC–MS for CO and CO_2_ contents. Ambient gas was also sampled from each jar before gas accumulation, after which 12 mL Nitrogen (N_2_) was injected into the jar to maintain the same air pressure. The sampling of gas was taken at intervals of around 7 days during the first month and then after 10 days until the end of the 60‐day incubation. Lid‐exchanging and sampling took place under a sterile cabinet. After gas sampling for GC–MS, the jars were placed in a 20‐L air‐tight plastic drum for conducting CO and CO_2_ emission measurements using the gas analyser in the same way as described above for the other experiments.

### 2.4 Microscopy Observations

Endosymbiont bacteria have been reported as existing both inside (i.e., intracellular) and outside (i.e., extracellular) fungal hyphae where they can affect fungal growth and phenology (Itabangi et al. 2022). Scanning electron microscopy (SEM), transmission electron microscopy (TEM) and light microscopy (LM) observations were therefore conducted to determine the possibility for the existence of extracellular and endosymbiont bacteria associated with the fungal hyphae.

For SEM and TEM, small pieces of actively growing mycelia were removed from agar plates (from the growing edge of the fungus) and fixed directly in 2.5% glutaraldehyde (v/v) containing 2.5% (v/v) paraformaldehyde in 0.1 M sodium cacodylate (pH 7.2) for 3 h at room temperature. Thereafter, the samples were washed (3 × 20 min) with the same buffer and subsequently post‐fixed in 1% (w/v) osmium tetroxide in 0.1 M buffer (pH 7.2) for 2 h RT. After washing in water (3 × 1 h, RT), samples were dehydrated in a progressive series of alcohol (20%–100% 20% steps) 15 min each. For SEM, the samples were subsequently dehydrated through an acetone series (1:3; 2:2, 3:1, pure acetone) 30 min each and critical point dried in a Polaron device using liquid CO_2_ as the transition fluid. Subsequently, the mycelia were mounted on stubs using carbon stabs, coated with Au using an Emitech K550X sputter device (Quorum Technologies Ltd., Ashford, Kent, UK) and examined using a Philips X30ESEM (Philips, Eindhoven, Holland; now Thermo Fisher Scientific) at 15–20 kV (Daniel et al. 2004). Images were recorded digitally.

For TEM and LM, mycelial samples after alcohol dehydration described above were embedded in London White Resin (London Resins Co Ltd., Basingstoke, UK) and the resin cured overnight in air‐tight gelatin capsules. Semi‐thin sections (ca. 1 μm) for LM and ultrathin sections (ca. 70–90 nm) for TEM were cut with a Reichert Ultracut E ultramicrotome using glass or diamond knives. For TEM, sections were collected on copper grids and observed unstained or after double staining with lead citrate (15 min) and 2% aq. (w/v) uranyl acetate (30 min) using a Philips CM12 (Philips, Eindhoven, Holland; now Thermo Fisher Scientific) TEM fitted with a LaB6 electron source at 60–100 kV. Images were recorded on Kodak 4489 negative film and films subsequently scanned using an Epson Perfection Pro 750 film scanner. For LM, sections mounted on glass slides were stained with 1% aq. (w/v) toluidine blue in 1% borax (pH 8.5). Sections were examined using a LM (Deltapix, Samourn, Denmark) equipped with an Infinity X‐32 digital camera.

### 2.5 Data Analysis

Data conversion of emitted gas concentration from ppm (*X*) to mg/m^3^ (*Y*) is performed using the following equation (Terrie and Boguski 2006):

(1) $$Y\left(\mathrm{mg}/m^{3}\right)=X\left(\mathrm{ppm}\right)\times \frac{M_{x}}{22.4}\times \frac{273}{273+T}\times \frac{P}{1013}$$

where *M*_*x*_ is the molecular weight of CO (28.01 g/mol) or CO_2_ (44.01); *T* is gas temperature (°C) and *P* is the atmospheric pressure (hPa).

The measured volumetric concentrations were then transformed to the total mass emitted per 100 g dry mass of initial substrate per day:

(2) $$\begin{array}{c} Z\left(\mathrm{mg}/d/100\;g\;\mathrm{IMS}\;\mathrm{DM}\right)=\left(Y_{\text{substrate}}-Y_{\text{ambient}}\right)\times V_{\text{container}}\\ \times \frac{60m\times 24\;h}{\text{accumulation time}\;\left(m\right)}\times \frac{100\;g}{\mathrm{IMS}\;\mathrm{DM}\;\left(g\right)} \end{array}$$

where *V*_container_ is the volume of the cylinder where the gas was accumulated for the measurement.

Accumulation of gas emission for 60 days' incubation was calculated by

(3) $$\mathrm{TZ}\left(\mathrm{mg}\right)=\sum_{t=1}^{t=60}\left(D_{t}-D_{t-1}\right)\times \left(Z_{t}+Z_{t-1}\right)/2$$

where *D*_*t*_ and *D*_*t*−1_ are Day *t* and *t* − 1, respectively.

The mass balance calculation was based on dry mass distribution between substrate, fruit bodies and estimated biomass losses due to emissions. The estimation of biomass loss due to carbon emission was calculated using Equation (4):

(4) $$\text{Biomass loss}\;\mathrm{due}\;\text{to emission of}\;{\mathrm{CO}}_{x}=\left(\mathrm{TZ}\times 12/M_{x}\right)\times C_{\text{substrate}}$$

where 12 and *M*_*x*_ correspond to the elemental weight of carbon and molecular weight of carbon‐monoxide or carbon‐dioxide; *C*_substrate_ refers to the content of carbon in the substrate that was determined using elemental analysis by the company Eurofins (https://www.eurofins.se/).

Correlations between CO and CO_2_ emissions from different substrate and fungi, as well as between the gas analyser and GCMS, were analysed for all replicates using the statistical software SPSS (IBM SPSS version 29.0). A Pearson test was used to determine the statistical significance of the correlations.

## 3 Results and Discussion

### 3.1 General Observations

On average and in all treatments with the inoculated fungi, the mycelia fully colonised the substrate after 20 ± 3 days of incubation for *P. ostreatus* and 30 ± 4 days for *L. edodes* and *P. enygii*. During the 60 days of the experiment period, fructification was observed in two treatments: *P. ostreatus* in Experiment 4 on Day 28 and *L. edodes* in 85° hot air treatment of Experiment 2 on Day 55 ± 4. The fruit bodies of *P. ostreatus* were harvested on Day 36 ± 3 and *L. edodes* on Day 68 ± 5. In addition, *L. edodes* grown on alder substrate (Experiment 8) started fructification on Days 64–67 and was harvested on Day 74 ± 3.

On all gas measurement occasions, the ambient oxygen concentration was around 20.6%–21.0% (v/v) and the oxygen in sampled gases 18.0%–20.9% (Table 1) indicating a slight decrease from ambient oxygen in all experiments and fungal growing stages. There was, in general, a slight increase in gas temperature in sampled gas compared with that in ambient gas (on average, 21.3°C–22.6°C vs. 21.7°C–22.9°C).

### 3.2 Fungal‐Genesis of Carbon Monoxide

**FIGURE 2:**
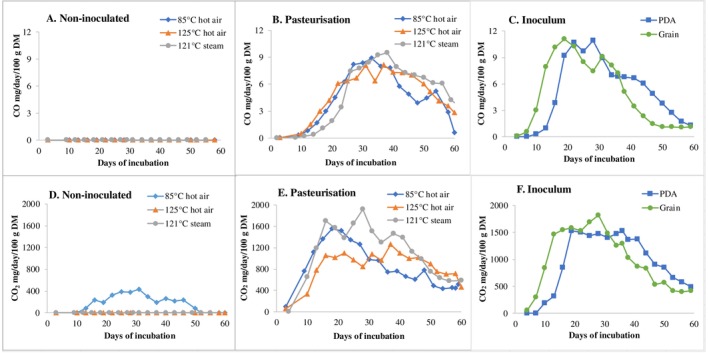
Emission of carbon monoxide (CO) (A–C) and carbon dioxide (CO_2_) (D–F) from different treatments, measured by Ecom‐J2KN gas analyser. Experimental settings are shown in Table 1. Data refer to mg gas emitted per day/per 100 g DM of initial substrate. Every point is the average of three repeated readings at 1, 1.5 and 2 min after starting measurements. Measurements were conducted by extraction using the gas analyser from a 20 L airtight drum containing four micro‐boxes of birch‐based substrate.

In Experiment 1 where no fungus was inoculated, CO emission was mostly non‐detectable regardless of pasteurisation regime and temperature (Figure 2A), except on Days 22 and 34 when negligible amounts of CO were measured (≤ 0.03 mg per day and per 100 g DM initial substrate; measured by gas analyser). In contrast, significant CO emissions were found in all pasteurised or sterilised treatments of Experiment‐2 when *L. edodes* was inoculated (Figure 2B).

The curve of CO emission against time (Experiment 2) generally showed a bell‐form and peak around 30–40 days after inoculation, i.e., when the mycelia had fully colonised the substrate and the observations on growth of shiitake fungus was obvious. At maximum, CO concentration in measured gas was around 9 mg/day/100 g DM initial substrate, corresponding to ca. 15 ppm/h/100 g DM in a 20‐l space, based on gas analyser measurements.

It is notable that the autoclaved initial substrate (in saturated steam at 121°C and 2 bar) resulted in slightly lower CO emissions during the first 3 weeks of incubation (Figure 2B). This coincides with our previous observations that autoclaving delays colonisation compared to hot‐air pasteurisation (Xiong et al. 2019). Hot‐air pasteurisation at 85°C resulted in relatively lower CO emissions during Days 40–54 compared to 125°C and autoclaving, prior to fructification.

As shown in Figure 2E,F, the formation of CO was paralleled by emissions of CO_2_, which also showed a bell‐form and association with the fungal growth pattern. The highest concentration of CO_2_ was about 2000 mg per 100 g initial substrate per day from autoclaved substrate. A detailed discussion on CO_2_ emission is given in Section 3.6.

**FIGURE 3:**
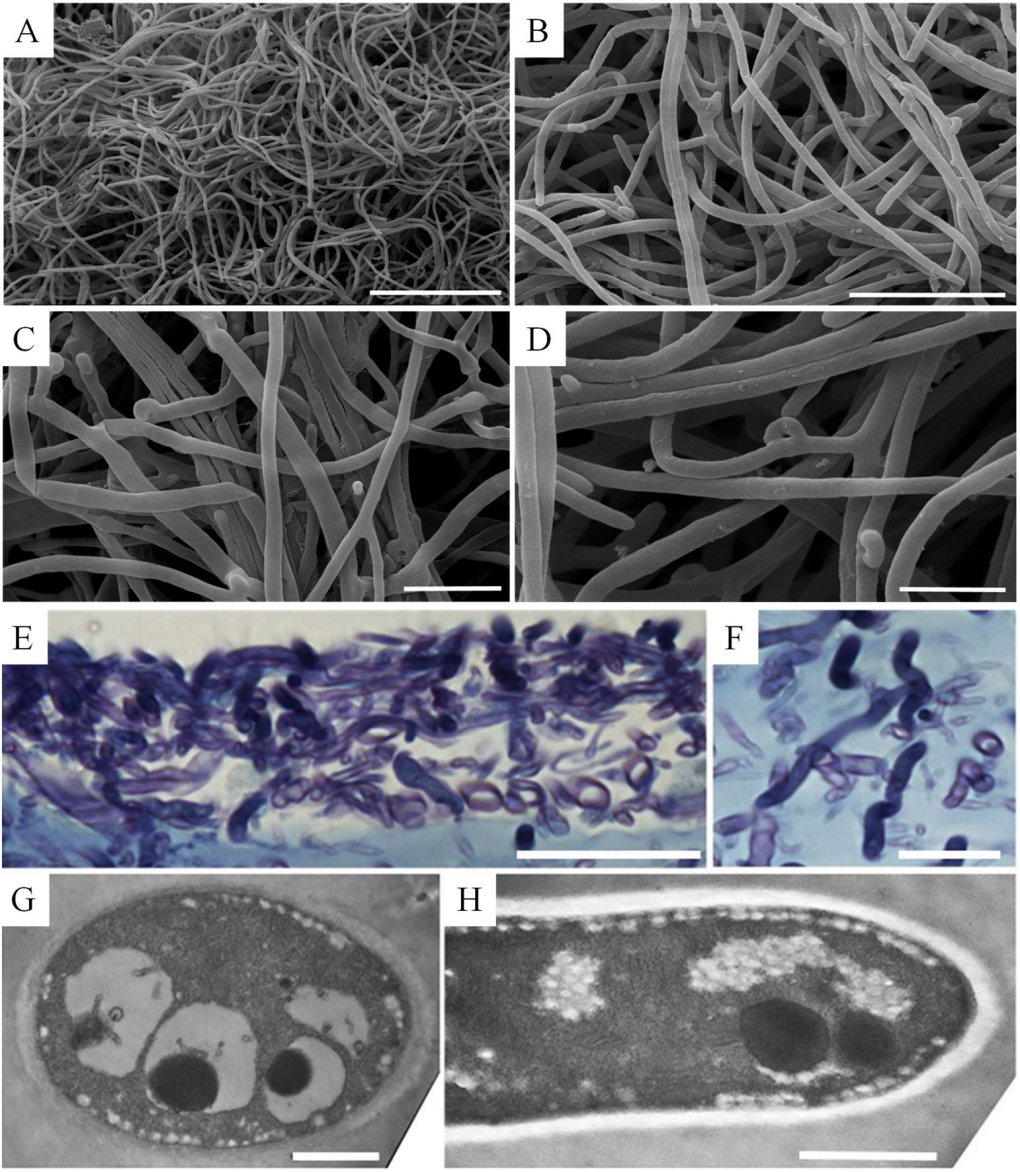
Microscopy images of shiitake mycelia from potato dextrose agar (PDA) inoculum. (A and B) Scanning electron microscopy (SEM) images from the original stock inoculum; (C and D) SEM images from sub‐cultured (2 weeks) samples. No surface evidence can be found for extracellular bacteria associated with the mycelia/hyphae. (E and F) LM images showing examples sectioned through shiitake mycelia grown on PDA, stained with 1% (w/v) toluidine blue. The sections are from sub‐cultured (2 weeks) samples and from actively growing fungi from the surface of the agar plate. Both deeply stained (dark purple) and light stained almost colourless hyphae are apparent. No surface evidence was found for the presence of extracellular bacteria associated with the mycelia/hyphae. (G and H) Transmission electron microscope (TEM) images showing examples sectioned through shiitake mycelia grown on PDA. The sections of representative individual sectioned hyphae show well characterised cellular ultrastructure. With the numerous sections and TEM observations made, no evidence for intracellular (i.e., or extracellular bacteria) within the cultured mycelia was noted. Bars: (A) 50 μm; (B) 20 μm; (C and D) 10 μm; (E) 10 μm; (F) 5 μm; (G) 1.0 μm and (H) 1.0 μm.

To examine the possible involvement of endosymbiont bacteria from grain spawn, a subculture of PDA inoculum was prepared and used for Experiment 3 using double autoclaved substrates. As shown in Figure 2C, there was little difference between using grain spawn and PDA inoculum in emitted CO quantity, although the PDA inoculated substrate caused a slight time lag. The PDA inoculum was composed of a ‘pure’ mycelia inoculum originating from several steps of sub‐culturing. Observations of the PDA inoculum using correlated LM, SEM and TEM confirmed that both the original and sub‐cultures did not show the presence of extracellular or intracellular bacteria associated with the mycelia (Figure 3).

This strongly suggests that cultivated *L. edodes* can independently generate CO, which may reflect a normal physiological activity of the fungus during growth. The delayed CO emission in PDA inoculated substrate (Figure 2C) likely reflected the slow mycelial growth caused by the lower density of mycelia and less nutrients in the PDA inoculum than with grain spawn.

In the 85°C pasteurised non‐inoculated substrate (Figure 2A,D), the observation that only very tiny traces of CO, whereas rather significant CO_2_ were detected, could be explained by gas emission from the parenchyma cells in the wood (Gessler 2017), although possibly it may also result from microbes that were not deactivated by 85°C heating.

### 3.3 Abiotic Influence on CO Emission From Shiitake

**FIGURE 4:**
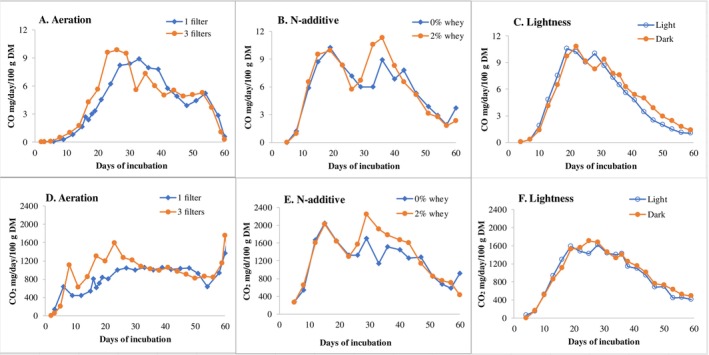
Influence of abiotic factor on CO and CO_2_ emission from *L. edodes*. Experimental settings are shown in Table 1. Data refer to mg gas emitted per day/100 g DM of initial substrate. Every point is the average of three repeated readings at 1, 1.5 and 2 min after starting measurements. Measurements were conducted by extraction using a gas analyser from a 20 L airtight drum containing four micro‐boxes of birch‐based substrate. (A and D) Aeration, (B and E) N‐additive and (C and F) Lightness.

The fact that growth of *L. edodes* was associated with the generation of CO suggests that any factors that can play a role on fungal growth could also affect CO emissions. Thus, three growth conditions were also studied (Figure 4). The CO emission patterns from the three experiments were similar to Experiment 2, showing bell‐form dynamics and peaking between Days 20 and 40 after fungal inoculation. The peak concentration of CO was 9–10 mg/day/100 g DM initial substrate when measured by gas analyser. CO_2_ emission peaked mostly between Days 20 and 30, at the time when the mycelia was at the full colonisation stage, with a ratio of between 1600 and 2200 mg/100 g substrate per day.

#### 3.3.1 Effect of Aeration Condition

The emission quantity and pattern were generally rather similar from shiitake growth boxes with 1 or 3 air‐exchanging filters (Figure 4A). The oxygen content in ambient gas was the same (i.e., 20.9%) for both treatments, and similar at 20.1% ± 0.3% and 20.4% ± 0.2% (mean ± standard error, *n* = 74 and 82, respectively) in the sampled gas for treatment with one and three filters, respectively. Enlargement of the air exchange openings from one to three increased slightly the CO emission especially after 20–30 days incubation, during which a more rapid mycelial colonisation was observed in the growth boxes with three filters. It is worth noting that even one aerobic filter equipped microbox has proved effective previously for such experiments (Chen, Martín, Finell, et al. 2022). Clearly, CO emission is unlikely generated under hypoxia conditions, in contrast to previous studies that indicate biogenesis of CO was enhanced by low oxygen environments (Siegel and Siegel 1987; Sobieraj et al. 2023 and references therein). Well aerobic conditions apparently increased the CO formation from shiitake.

#### 3.3.2 Effect of Nitrogen Addition

Addition of 2% (w/w) whey slightly increased CO emission during Days 30–40 of incubation (Figure 4B). Nitrogen is important for mushroom growth and fruit body production. Whey is known to be an easily absorbed nitrogen source for shiitake growth (Chen, Martín, Finell, et al. 2022). Added whey was found to increase shiitake's carbohydrate degradation, slightly prolong the life cycle and delay fructification (Chen, Martín, Finell, et al. 2022), which may explain the increase in CO emission.

#### 3.3.3 Effect of Illumination

**FIGURE 5:**
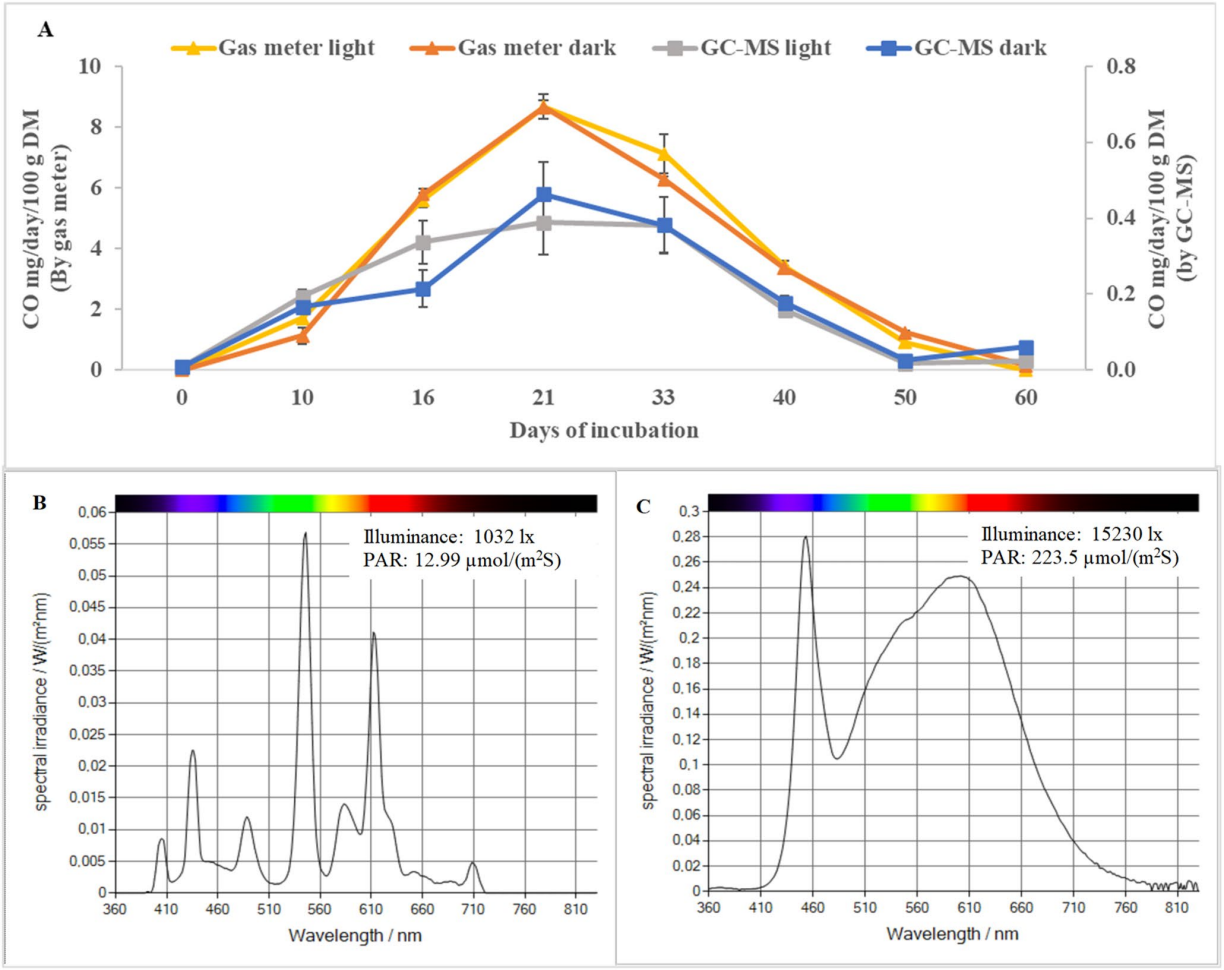
Similarities in CO dynamics over 60 days of incubation using two measuring methods (A) and spectra of LED lights below and above the fungal cultivation flasks for the light treatment (B and C, respectively). No significant difference was found between light and dark in CO emission (*p* > 0.05). PAR: Photosynthetically Active Radiation. All data are from and for the Experiment 6b. Spectral diagrams were generated by the light metre and inbuilt software (Gigahertz‐Optik, Germany).

Figures 4C and 5A show almost equal quantities and similar dynamic patterns of CO emissions from shiitake substrates placed in either light‐ or dark conditions during the incubation. Notably, significant differences in Figure 5 were due to the two different measurement methods (see Section 3.8 for further elaborations). Treatment with light was by constant visible light (i.e., both day and night; Figure 5B,C), which has normally been considered stressful for fungal mycelia (Fuller et al. 2015). The lowest light intensity was 12.99 μmol/m^2^s (1032 lx), which was even stronger than that suggested for optimization of shiitake fructification (Abe and Nishizawa 2011; Miyazaki et al. 2011; Katagiri et al. 2019). Nevertheless, there was no difference found between light‐ or dark conditions in CO emission associated with shiitake mycelia growth in this study. This contrasts with earlier reports by Westlake et al. (1961) and Siegel and Siegel (1987) that suggested CO be emitted by fungi under dark conditions.

Darkness has been considered necessary for mycelial growth at early stages after inoculation (Katagiri et al. 2019). Light exposure was shown to be important for mushroom fruiting and pigmentation (Leatham and Stahmann 1987) and regulation of fungal biological processes (Kim et al. 2020; Fuller et al. 2015). Therefore, both light and dark represent fundamental environmental conditions like aeration, temperature and nutrient conditions. Thus, it is reasonable that CO emits regardless of growth under light‐ or dark conditions.

### 3.4 Impact of Substrate Variety and Fungal Species

**FIGURE 6:**
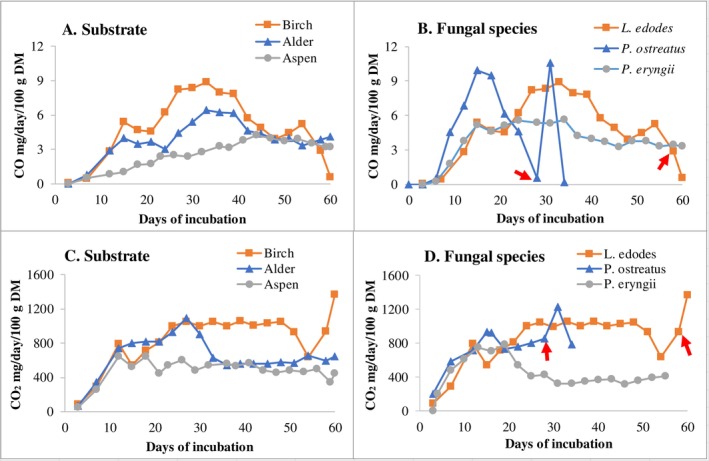
Variations in CO and CO_2_ emission from *L. edodes* with substrate and fungal species. Experimental settings are shown in Table 1. Data refer to mg gas emitted per day and per 100 g DM of initial substrate. Every point is an average of three repeated readings at 1, 1.5 and 2 min after starting. Measurements were conducted by extraction using a gas analyser from a 20 L airtight drum containing four micro‐boxes of fungal growing wood substrate. Arrows indicate the time when pins were observed. (A and C) Substrate and (B and D) fungal species.

Substrate type had one of the largest effects on CO emission from *L. edodes* (Figure 6A) compared with other experiments in this study. The birch‐based substrate produced the highest CO emission, followed by alder‐ and aspen‐based substrates.

The fungal species also significantly influenced CO and CO_2_ emissions. *P. ostreatus* had a short life cycle (36 days) and generated in total less CO and CO_2_ than *L. edodes* and *P. enrygii* (Figure 6B,D). Although growing on the same birch substrates, *L. edodes* produced significant larger amounts of CO and CO_2_ than *P. enrygii* during the 60 day incubation period.

In the present study, the particle sizes of different substrates were generally the same (≤ 5 mm). It is understandable that different hardwoods may vary with chemical compositions, which may form inconsistent microenvironments for fungal growth. The responses of any fungus to different environments can be variable, resulting in variations in fungal metabolic activities and growth.

However, depending on genetic differences formed during evolution, fungal species are expected to show different responses to similar environments, which is what we observed with CO and CO_2_ emissions between the fungal species used (Figure 6).

### 3.5 Correlation Between CO and CO_2_

CO_2_ emission patterns from this study agree with Donoghue and Denison (1995) who also found variations according to fungal strain/species, physiological growth stage and growing conditions.

Significant correlations between CO and CO_2_ were found with most treatments with inoculated fungi. The correlations can be depicted as showing a semi‐parabola curve, in which CO_2_ increases quadratically with increasing CO emission. In only two cases were the correlations insignificant: i.e., *L. edodes* growing with aspen and *P. eryngii* on birch, which was consistent in a repeated run. The exact reasons for this are unknown (Table 2).

**TABLE 2:** Correlation between CO and CO_2_ from fungi in different treatments.

Experiment	Treatment	*R* ^2^	Relation	*N*	*p* (2‐tailed)
1. Non‐inoculated	85°C hot air	—	—	—	—
	125°C hot air	—	—	—	—
	121°C steam	—	—	—	—
2. Pasteurisation regime	85°C hot air	0.754	Quadratic	22	< 0.01
	125°C hot air	0.774	Quadratic	24	< 0.01
	121°C steam	0.887	Quadratic	19	< 0.01
3. Inoculum type	Grain	0.942	Quadratic	20	< 0.001
	PDA	0.970	Quadratic	20	< 0.001
4. Aerobic availability	1 filter	0.751	Quadratic	22	< 0.01
	3 filter	0.540	Quadratic	21	< 0.05
5. Nitrogen addition	Whey 0%	0.802	Quadratic	17	< 0.01
	Whey 2%	0.822	Quadratic	17	< 0.01
6a. Illumination	Light	0.975	Quadratic	20	< 0.001
	Dark	0.963	Quadratic	20	< 0.001
6b. Illumination (for GA vs. GC–MS)	Light—GA	0.919	Quadratic	24	< 0.001
	Light—GC–MS	0.699	Quadratic	24	< 0.01
	Dark—GA	0.891	Quadratic	24	< 0.001
	Dark—GC–MS	0.835	Quadratic	24	< 0.001
7. Substrate types	Birch	0.751	Quadratic	22	< 0.01
	Alder	0.544	Quadratic	21	< 0.05
	Aspen	0.463	Quadratic	21	ns
8. Fungal species	*L. edodes*	0.751	Quadratic	22	< 0.01
	*P. ostreatus*	0.698	Quadratic	11	< 0.05
	*P. eryngii*	0.359	Quadratic	19	ns

### 3.6 Carbon Emission and Mass Balance

In a further study, a mass balance assessment was performed by continuing measurement of CO and CO_2_ for the entire life cycle of shiitake and grey oyster mushrooms. As shown in Table 3, the contribution of CO_2_ emission from the first 60 days was estimated as 84%–91% of the total emission from entire life cycle.

**TABLE 3:** Mass balance of the entire life cycle of *L. edodes* and *P. ostreatus* growing on wood substrates. The calculation of biomass loss due to emissions is based on Equation (4) using measurements from the gas analyser.

	Unit	*L. edodes*	*L. edodes*	*P. ostreatus*
		Alder	Birch	Birch
Day to harvest	Day	74.30 ± 3.03	64.00 ± 4.93	36.33 ± 3.20
Initial substrate mass	g DM	100	100	100
Spent substrate mass	g DM	62.24 ± 0.29	59.33 ± 1.40	70.96 ± 0.66
Dry fruit body mass	g DM	5.76 ± 0.57	5.87 ± 0.76	7.06 ± 0.53
Biomass loss due to CO_2_	g DM	23.54 (19.83)^a^	29.36 (26.81)	14.98
Biomass loss due to CO	g DM	0.19 (0.17)	0.27 (0.25)	0.12
Unknown losses^b^	g DM	7.86	5.17	6.86
Biomass ratio CO/CO_2_	%	0.81	0.92	0.80

^a^ Data in brackets are for 60 days of incubation.

^b^ Estimated by differences.

The biomass loss from CO emission was around 0.8%–0.9% of that from CO_2_ emission, which is 8–9 times higher than that reported by Hellebrand and Schade (2008) who observed a ratio of emitted CO‐carbon to CO_2_‐carbon between 0.1‰ and 1‰ from decomposing organic wastes and litter.

### 3.7 Gas Analyser (GA) vs. GCMS for Measuring Carbon Emission

There were strong positive correlations between the gas analyser and GCMS methods for the measurement of both CO and CO_2_ (Table 4). However, differences in measurements were also significant, with the values obtained from the gas analyser much larger than those from the GC–MS method. Generally, the absolute values differed by factors of 1.7–1.9 for CO_2_ and 19–22 for CO. The differences cannot be fully explained by the current study but are somehow predicted. Both sampling and principles are different between the two methods. With the gas analyser the emitted gas was sucked by a pump under pressure (300–1100 hPa) and then detected by an infrared prob., whereas with the GCMS method, the emitted gas was sucked manually using a medical syringe and analysed via gas chromatography–mass spectrometry. However, the achieved strong correlations between the two methods suggest that the portable gas analyser can be used even for CO measurements, but the values should be converted to a value that can be comparable to a more reliable method such as GC–MS. GC–MS methods are widely used as a reliable approach to identify and quantify gas emissions, while portable gas analysers or probes are used more often in industrial practices and environmental monitoring. Our findings will help future industrial monitoring of gases by using gas analyser.

**TABLE 4:** Correlations between the gas analyser (GA) and GC–MS methods in measuring CO and CO_2_ based on the data from Experiment 6b.

	Light		Dark	
	CO	CO_2_	CO	CO_2_
*n*	24	24	24	24
*R* ^2^	0.701	0.851	0.657	0.815
*P*_sig_	< 0.001	< 0.001	< 0.001	< 0.001
Regression (*y* = GA reading, *x* = GC–MS values)	*y* = −22.672*x* ^2^ + 27.017*x* − 0.027	*y* = 1.910*x* + 69.491	*y* = −19.718*x* ^2^ + 25.942*x* − 0.116	*y* = 1.742*x* + 14.358

Both the portable gas metre and GC–MS method confirmed positive CO emissions from fungi in this study. However, the divergences of gas measurement quantities between the two methods suggest more advanced technology is urgently needed. More sensitive and effective analyses on the carbon emission are important for future academic research, carbon modelling and practical monitoring of working environment.

### 3.8 Potential Mechanisms of Fungal CO Emission

Several dominant theories have been proposed to explain CO generation during biomass decomposition under aerobic conditions. In this study we evaluated three such mechanisms considering our findings.

*CO*_*2*_ *reduction* via *CO dehydrogenase*. Sobieraj et al. (2023) reviewed CO production pathways and proposed that the involvement of the bacterial enzyme CO dehydrogenase (COHD) was one of the major mechanisms. COHD catalyses the reduction of CO_2_ to CO following the reaction CO_2_ + H_2_ → CO + H_2_O. This enzyme is known to exist in methanogenic, acetogenic and sulphate‐reducing bacteria under both aerobic and anaerobic conditions (Sobieraj et al. 2023 and references therein). However, our results contradict the involvement of bacterial CODH. In Experiment 3, where strong CO–CO_2_ correlation was observed, the substrates were double autoclaved and inoculated with sub‐cultured PDA that was free of bacterial contamination, as confirmed by LM, SEM and TEM (Figure 3). CO emission occurred under fully aerobic, bacteria‐free conditions, suggesting that CO production was independent of bacterial COHD. However, it remains an open question whether there are fungal COHD‐like enzymes or analogues involved.

*CH*_*4*_ *oxidation theory*. Photochemical oxidation of methane (CH_4_) and non‐methane hydrocarbons is considered a major natural source of atmospheric CO (McConnell et al. 1971; Brenninkmeijer and Novelli 2003). However, CO production in our experiments occurred under both light and dark conditions (Experiment 6; Figures 4C and 5), which is inconsistent with a light‐driven photochemical process. An alternative version of this theory, proposed by Haarstad et al. (2006), links CO formation to CH_4_ presence and O_2_ reduction under aerobic conditions, potentially due to the oxidation by methanogens. Nevertheless, bacterial involvement is unlikely in our study, especially in the PDA inoculum treatments where microscopy also confirmed the absence of bacteria. Although we attempted CH_4_ measurements, data were inconclusive due to technical limitations. Further investigations into possible CH_4_–CO interactions in fungi are warranted.

*Thermochemical oxidation of biomass*. Previous studies (Hellebrand and Schade 2008; Stegenta‐Dabrowska et al. 2019) have shown that CO can form abiotically from heat‐treated biomass, independent of microbial activity, especially under high temperature and moisture conditions. CO emission was promoted by increased temperature, substrate wetness and oxygen supply. Alakoski et al. (2016) and Siwale et al. (2024) also reported that the oxidation of organic components in dried wood can generate CO, but additions of antioxidants substantially reduced its emission. However, in our Experiment 1, where non‐inoculated substrates were heat‐treated (85°C–125°C or autoclaved at 121°C), no significant CO was detected—suggesting that temperature alone does not account for fungal CO generation during fungal growth. In contrast, CO emissions in Experiment 2 did vary with pasteurisation temperature during active fungal growth, implying a possible interaction with fungal metabolic processes. It is well established that lignin degradation is a key oxidative process in fungal metabolism, mediated by lignin‐modifying enzymes such as laccases and lignin/manganese peroxidases. Whether these enzymatic activities contribute to CO formation remains to be further investigated.

In summary, our findings do not fully support any of the three conventional theories. Instead, they point towards a fungal‐specific metabolic origin of CO emission. The exact biochemical mechanism remains to be explored in future studies. One hypothesis is that the edible fungi might also contain haem oxygenase that can catalyse iron acquisition by degrading haem from decaying bio‐resource while releasing CO as a metabolic byproduct at the same time. Haem oxygenase has been found in fungal species such as *Saccharomyces cerevisiae* (baker's yeast) and *Schizosaccharomyces pombe* (fission yeast) (Kornitzer and Roy 2020; Mourer et al. 2019; Streng et al. 2021). It would be interesting to understand how CO‐metabolism correlates with mushroom fructification.

## 4 Conclusions

This study provides robust evidence that cultivated edible fungi—including *L. edodes*—can produce carbon monoxide (CO) during growth, independent of microbial contamination and regardless of light or oxygen availability. CO emissions closely mirrored the physiological growth curve of the fungi, with levels peaking during active mycelial colonisation. These dynamics paralleled CO_2_ emissions and were consistently observed across multiple species, substrates and growing conditions.

Microscopy (LM, SEM and TEM) confirmed the absence of intracellular or extracellular bacteria in the fungal cultures, reinforcing the conclusion that CO generation originates from the fungi themselves. Notably, while abiotic factors such as aeration, nitrogen supplementation and illumination had relatively minor effects, substrate type and fungal species significantly influenced CO and CO_2_ release. Among the tested conditions, shiitake grown on birch exhibited the highest emission rates.

Despite the consistent CO–CO_2_ correlation, the underlying biochemical mechanism remains unresolved. Several theoretical pathways—including microbial CO dehydrogenase activity, CH_4_ oxidation and thermochemical oxidation—were considered, yet none fully explain the observed CO generation under sterile and aerobic conditions. This points to the possibility of an unrecognised fungal metabolic route, warranting further investigation.

While several known pathways of CO generation—microbial enzymatic, photochemical oxidation and thermochemical reactions—were considered, our evidence supports a fungal metabolic origin, independent of these established mechanisms.

Given CO's known toxicity and its role as an indirect greenhouse gas, our findings have important implications for occupational safety in mushroom cultivation and for broader carbon cycle modelling. Understanding fungal CO production is not only relevant for food production systems but may also reveal previously unaccounted sources of atmospheric CO, particularly in closed ecosystems or industrial settings.

## Author Contributions

**Shaojun Xiong:** conceptualization, project administration, funding acquisition, data curation, formal analysis, writing – original draft, writing – review and editing. **Geoffrey Daniel:** conceptualization, experiment design, investigation, data curation, writing – original draft, writing – review and editing. **Jannik Demuth:** investigation and data curation. **Mohsen Parchami:** investigation. All authors have read and approved the final version of this work.

## Funding

This work was supported by Svenska Forskningsrådet Formas (2022‐02404, 2022‐02760), Ekhagastiftelsen (2022‐39), VINNOVA (2017‐02705) and Kempestiftelserna (JCK22‐0028).

## Ethics Statement

The authors have nothing to report.

## Conflicts of Interest

The authors declare no conflicts of interest.

## Data Availability

The authors confirm that the data supporting the findings of this study are available within this article. After publication, the data may also be deposited at Svensk Nationell Datatjänst (SND) portal (https://researchdata.se/). Detail information can be available upon request.
